# Long Term Outcomes of Surgical Excision of Giant Papillae with Mitomycin C and Amniotic Membrane Transplantation in the Treatment of Refractory Palpebral Vernal Keratoconjunctivitis

**DOI:** 10.3390/medicina58010019

**Published:** 2021-12-23

**Authors:** Moushmi Patil, Jodhbir S. Mehta

**Affiliations:** 1 Cornea and External Eye Disease Department, Singapore National Eye Centre, Singapore 168751, Singapore; patil.moushmi@gmail.com; 2 Singapore Eye Research Institute, Singapore 169856, Singapore; 3 Ophthalmology and Visual Sciences Academic Clinical Program, Duke-NUS Graduate Medical School, Singapore 169857, Singapore

**Keywords:** vernal keratoconjunctivitis, giant papillae, amniotic membrane transplantation, mitomycin C

## Abstract

*Background and Objectives*: To report the long-term outcomes of patients with refractory Vernal Keratoconjunctivitis (VKC) who underwent surgical excision of giant papillae (GP) with mitomycin C (MMC) 0.02% and amniotic membrane transplantation (AMT). *Materials and Methods*: This is a retrospective interventional single-center case series including five eyes of four patients who had refractory, symptomatic VKC with GP, along with corneal shield ulcers and/or punctate epithelial erosions. They underwent surgical excision of GP with MMC 0.02% alone (1 eye) or with MMC 0.02% and AMT (4 eyes). Their long-term visual and surgical outcomes were studied. *Results*: All subjects were male with bilateral involvement and mean age of presentation 9.8 years. The surgery was uneventful in all cases. Amongst the four eyes which underwent MMC with AMT, only one eye demonstrated papillary regrowth requiring repeat surgery. Postoperative follow-up ranged from 59 to 77 months (median 66 months). Four patients had the best corrected visual acuity (BCVA) >/= 6/9.5. One patient had BCVA 6/15 at the final follow-up due to the presence of anterior corneal stromal scar and poor ocular surface. *Conclusions*: Surgical excision of GP in combination with MMC and AMT, in refractory VKC, is a good treatment option with better clinical outcomes over a longer follow-up.

## 1. Introduction

Vernal keratoconjunctivitis (VKC) is a chronic, bilateral, allergic inflammation of the ocular surface, which predominantly involves the upper tarsal and/or limbal conjunctival surface [1,2]. Seasonal exacerbations are common [3], however, a chronic perennial form was also described [4]. Typically, it was considered a type 1 hypersensitivity reaction, usually found in individuals or families with a history of atopy, with increased serum levels of total and specific IgE [5,6]. However, complex non-IgE-mediated mechanisms were described [1,7] with numerous inflammatory mediators detected in the tears of patients with VKC [8].

It is most commonly seen in the first decade of life with the mean age of onset 7.4 +/− 6.9 years and male preponderance [9,10]. Giant papillae (GP) greater than 1 mm in size over the upper tarsal conjunctiva (cobblestone appearance) is a common clinical manifestation of VKC [11]. With time, the size of the GP positively correlates with the persistence or worsening of the symptoms [12]. GP can cause pseudoptosis and corneal complications including punctate keratitis and shield ulcers [13]. Shield ulcers can result in decreased visual acuity due to scarring of centrally located ulcers, delayed re-epithelization, and superimposed microbial keratitis [14,15]. In severe VKC, pentacam can aid in the detection of keratoconus. Severe VKC can lead to permanent visual loss in children [16], and therefore it is essential to have a multidisciplinary approach closely collaborated amongst the ophthalmologist, pediatrician, and allergist [17].

The standard of care in the management of GP in VKC includes the use of topical antihistamines [18], mast cell stabilizers [19], topical immunomodulators [20], and intralesional [21], topical or systemic steroids [22]. In cases refractory to medical management, surgical excision of the GP alone or in combination with procedures like autologous conjunctival transplantation, intraoperative mitomycin C (MMC) 0.02%, cryoablation, or amniotic membrane transplantation (AMT) were described [23,24,25,26].

In this study, we present the long-term outcomes of five eyes of four patients with GP in severe VKC that failed conventional medical treatment and later underwent surgical excision of GP with MMC and AMT.

## 2. Materials and Methods

This study is a retrospective review of medical records of 4 patients (5 eyes) with refractory, symptomatic VKC with GP who underwent surgical excision of GP with MMC 0.02% alone (1 eye) or with MMC 0.02% and AMT (4 eyes) in the Cornea and External Eye Disease Department of Singapore National Eye Centre.

The diagnosis of VKC was made clinically, based on the presence of a history of chronic, bilateral, seasonally exacerbated conjunctivitis with symptoms of itching, redness, and mucoid discharge, with characteristic GP on the upper tarsal conjunctiva.

All eyes had received prior conventional medications for VKC, including topical antihistamines, topical mast cell stabilizers, topical and supra tarsal steroids, topical immunomodulators (cyclosporine or tacrolimus). As a result of refractory symptomatic GP failing conventional medications, all patients consented to surgical option—resection of GP with MMC and AMT.

## 3. Surgical Technique

**Figure 1:**
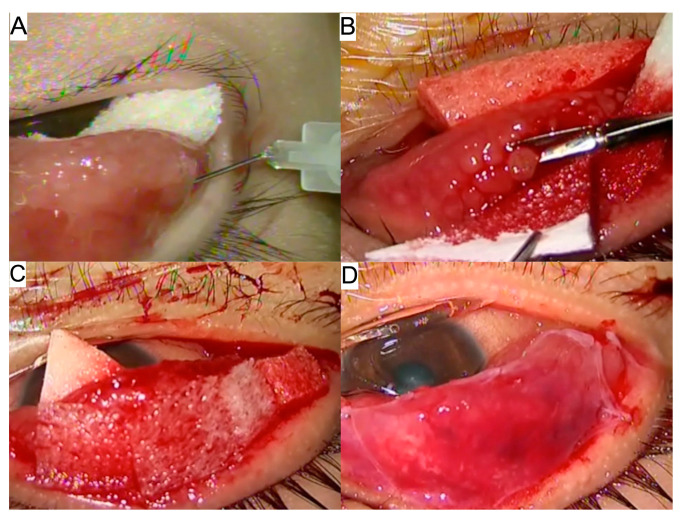
Surgical technique of giant papillary resection with Mitomycin C 0.02% and Amniotic membrane transplantation. (**A**) After everting upper eyelid, subconjunctival lignocaine and adrenaline were injected at the base of giant papillae. (**B**) Excision of giant papillae at the base was performed with Wescott scissors. (**C**) Weck cell sponge soaked in MMC 0.02% was applied onto tarsus for 5 min. (**D**) Amniotic membrane was placed over the tarsal surface with fibrin glue and sutured with 10-0 vicryl.

All surgeries were performed by a single surgeon under general anesthesia. The upper eyelid was everted to expose the tarsal conjunctiva. Subconjunctival lignocaine and adrenaline were injected at the base of the papillae. Excision of papillae at the base was performed with Wescott scissors. Weck cell sponge soaked in MMC 0.02% was applied onto tarsus for 5 min, followed by irrigation with a copious balanced salt solution. Amniotic membrane transplant was then applied to the tarsal surface with fibrin glue and sutured with 10-0 vicryl (Figure 1A–D).

Subconjunctival cefazolin, gentamicin, and dexamethasone were injected at the end of surgery and a bandage contact lens was applied to the eye.

## 4. Results

**Figure 2:**
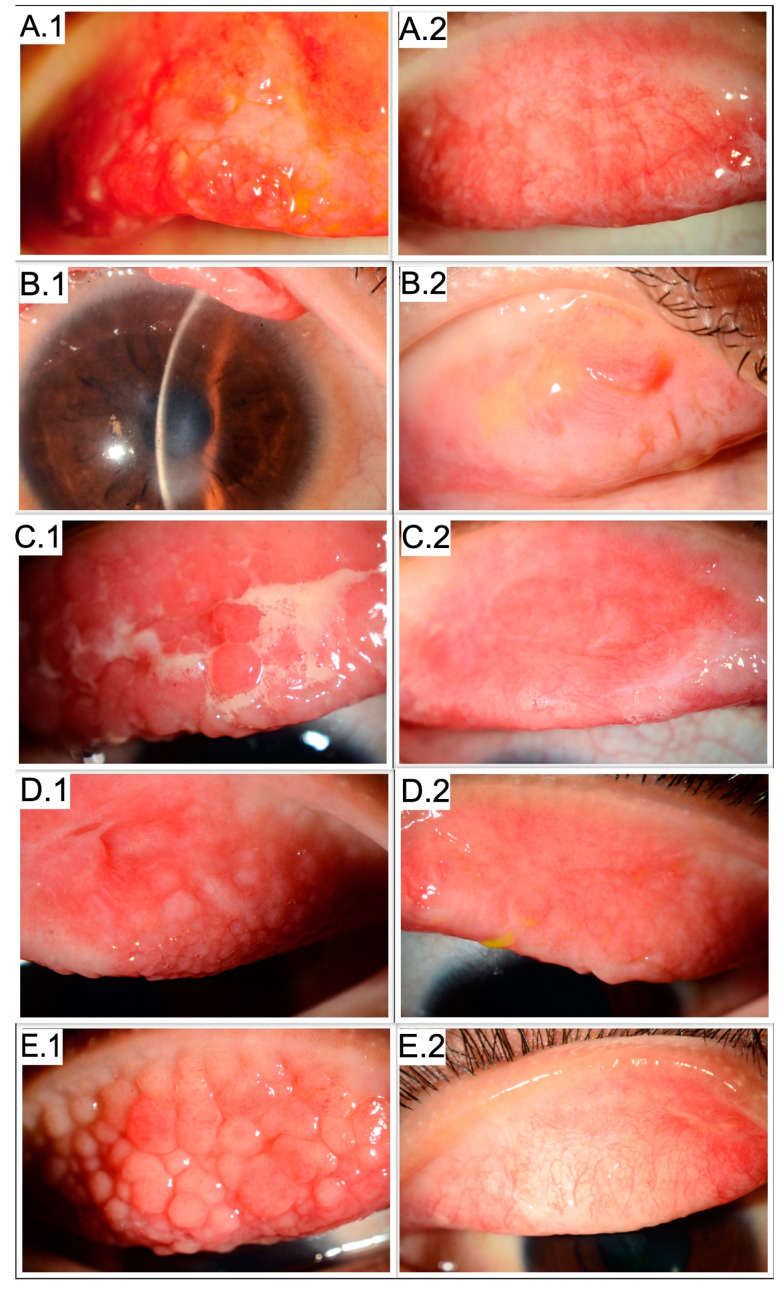
Pre- and Postoperative images of 4 patients (5 eyes) with vernal keratoconjunctivitis (**A.1**) Preoperative image showing giant papillae on upper tarsal conjunctiva (Case 1) (**A.2**) Postoperative image 6 weeks after surgery showing scarring of upper tarsal conjunctiva (Case 1) (**B.1**) Preoperative image showing anterior corneal stromal scarring secondary to previous shield ulcer (Case 2) (**B.2**) Postoperative image 8 months after surgery showing scarring of upper tarsal conjunctiva (Case 2) (**C.1**) Preoperative image showing giant papillae on upper tarsal conjunctiva with a mucoid discharge—right eye (Case 3) (**C.2**) Postoperative image 5 weeks after surgery showing scarring of upper tarsal conjunctiva—right eye (Case 3) (**D.1**) Preoperative image showing recurrence of giant papillae on upper tarsal conjunctiva and scarring 7 weeks after first surgery—left eye (Case 3) (**D.2**) Postoperative image 4 months after second surgery showing resolution of giant papillae and scarring of upper tarsal conjunctiva—left eye (Case 3) (**E.1**) Preoperative image showing giant papillae on upper tarsal conjunctiva (Case 4) (**E.2**) Postoperative image 6 weeks after surgery showing scarring of upper tarsal conjunctiva (Case 4).

This study included five eyes of four patients. All were male, with a mean age of 9.8 years (range 7 to 14 years) at presentation. Table 1 shows the patient demographics, preoperative and postoperative visual acuity, corneal complications, and follow-up duration. Table 2 shows the type of surgeries performed in all patients. All had bilateral involvement. Despite this, bilateral surgery for GP was required in only 1 patient. The surgery was uneventful in all cases. Figure 2A–E shows the preoperative and postoperative images of all patients. Postoperative follow-up ranged from 59 to 77 months (median 66 months). Amongst the 4 eyes which underwent AMT with MMC, only 1 eye demonstrated papillary regrowth requiring repeat surgery after 15 weeks {Case 3-left eye}. 4 patients had best corrected visual acuity (BCVA) >/= 6/9.5. One patient had BCVA 6/15 at final follow-up due to the presence of anterior corneal stromal scar and poor ocular surface {Case 2}. Case 1 had no exacerbation of symptoms in the right eye till the last follow-up, which was 8 years after surgery. Case 2 developed an acute flare in the right eye 2 years after surgery, which subsided with 4 months of topical steroid (prednisolone acetate 1% eye drops) treatment in tapering doses. Later the patient was prescribed ciclosporin 0.5% eye drops 2 times/day and olopatadine 0.1% eye drops 2 times/day. The eye was quiescent for the last 3 years and 10 months. Case 3 developed an acute flare of VKC symptoms 4 years and 10 months after surgery in the right eye and 4 years and 7 months after repeat surgery in the left eye. He was given topical steroids (dexamethasone 0.1% eye drops) in tapering doses for 4 months in the right eye and 9 months in the left eye. Thereafter, he was placed on tacrolimus 0.03% ointment 2 times/day and olopatadine 0.1% eye drops 2 times/day for both eyes. The right and left eyes were quiescent for the last 9 months and 4 months, respectively. Case 4 developed steroid-induced glaucoma and underwent trabeculectomy 3 months after surgery. He experienced an acute flare of VKC symptoms 1.5 years after surgery which subsided after 2 months with tapering doses of prednisolone 0.1% eye drops. He was subsequently prescribed ciclosporin 0.1% eye drops 2 times/day, olopatadine 0.1% eye drops 2 times/day, and sodium cromoglycate 2% eye drops 4 times/day. His eye was quiescent for the past three years and five months.

## 5. Discussion

Vernal Keratoconjunctivitis is an allergic chronic and recurrent inflammatory disease of the cornea which tends to have a long disease course with short intervals between relapses [6,27]. The visual impairment caused by the complications can affect daily activities and schooling since most of the affected patients fall in the school-going age group [10]. Based on the disease severity, treatment is started in a step-wise manner [28]. Initial treatment for the milder disease is with topical antihistamines and mast cell stabilizers [18]. Topical corticosteroids are prescribed for exacerbations, but their use is limited to the acute phase of the disease due to their side effects [29,30]. Topical immunomodulators like cyclosporin and tacrolimus have also been found to be effective to alleviate signs and symptoms in severe VKC [20,31].

The current treatment options for VKC refractory to medical therapy are simple scraping of the base and margins of the ulcer, with the removal of the inflammatory material (i.e., the plaque), which aid in rapid re-epithelialization [22]. However, the presence of the mechanical stimulus, from the GP, can result in recurrences of punctate epitheliopathy and corneal shield ulcers. Removal of the mechanical stimulus along with suppression of the immunological process of the disease can help prevent disease recurrence. Surgical excision of the GP can be combined with procedures like autologous conjunctival transplantation, application of intraoperative MMC, cryotherapy, or AMT alone [23,24,25,26]. Table 3 shows the various surgical procedures with their outcomes in refractory VKC patients.

Surgical resection with cryotherapy can result in irregular conjunctival scarring and surface irregularities of the superior palpebral conjunctiva [32]. Jiang D et al. reported that resection and cryotherapy combined with AMT were found to be effective for VKC with GP [25] but recurrences of GP were seen in 12.5% of the cases in their study. Autologous conjunctival transplantation from the inferior conjunctiva after surgical resection of upper tarsal GP was tried by Nishiwaki–Dantas et al. [23]. However, this procedure only removed the mechanical insult from the GP without having any influence on the immunological process of the disease. Therefore, postoperatively, patients were treated with topical mast cell stabilizers, antihistamines, or anti-inflammatory agents. Recurrence of the papillae was observed on the inner margin of the graft (not on the graft) in 16.6% of the cases over a follow-up period of 9 to 27 months. Studies were described using MMC alone [24] and AMT alone [26] after resection. The recurrence of GP observed with MMC alone was 57.1% with a postoperative follow-up period of 12–18 months [24]. MMC-related complications, e.g., ocular discomfort, eyelid erythema, corneal epithelial defects, corneal and scleral melting were reported [33,34,35]. No recurrence of GP was seen when AMT was used alone after resection but an exacerbation of VKC symptoms was seen in 69.2% of cases over a follow-up period of 14.2 +/− 4.2 months [26]. The longest follow-up period for the above studies ranged from 15.7 to 27 months (median 18 months).

Our series included patients with GP that were associated with corneal complications like coarse punctate keratopathy and shield ulcers unresponsive to medical treatment including topical steroids, topical immunomodulators, topical antihistamines, topical mast cell stabilizers, and/or supra tarsal steroid injection. In our study, AMT was combined with the application of MMC 0.02% over the resected papillae. The amniotic membrane itself has an immuno-suppressive effect [36] and transplantation of the amniotic membrane can avoid damage to the coarse surface of the conjunctiva of the upper eyelid after surgery. Suppression of fibroblast proliferation by MMC can result in a reduction of activated eosinophils, which helps inhibit postoperative inflammation and regrowth of papillae, preventing further complications [24]. The side effects of our surgical technique could hypothetically include complications of MMC, [33,34,35] recurrence of the disease [24], and microbial infections after AMT [37]. Commercially available gamma irradiated AMT, may help limit the latter, but this warrants further investigation [38]. In this study, with a postoperative follow-up ranging from 59 to 77 months (median 66 months) which is the longest follow-up case series reported till date, recurrence of GP was seen in one eye. The corneal punctate epitheliopathy as well as the corneal shield ulcers healed after surgery. MMC-related complications and microbial infections secondary to AMT were not observed. One case had no exacerbation of symptoms after surgery till his last follow up of 8 years (Case 1). Four eyes (Cases 2, 3, and 4) had one episode of exacerbation of VKC symptoms 18 to 58 months (median 39.5 months) after surgery with no corneal complications and were managed medically. They were treated with tapering doses of topical steroids for 2 to 9 months (median 4 months) depending upon their severity. Later, they were placed on topical mast cell stabilizers and/or topical antihistamines and topical immunomodulators. Case 3 underwent repeat surgery for GP recurrence in the left eye 15 weeks after the first surgery. Cases 2, 3, and 4 had residual corneal scarring secondary to shield ulcer. Case 3 developed inferonasal symblepharon in both eyes which did not affect ocular motility. Minimal to moderate subtarsal scarring of the upper eyelid was seen in all eyes.

## 6. Conclusions

Due to the uncommon nature of this severe form of VKC, it is difficult to get a large patient cohort. With the limitation of a small case series, surgical excision of GP with a combination of MMC and AMT, in refractory VKC with failed conventional medical treatment seems to have good clinical outcomes over a longer follow-up period. However, long-term prospective studies with a larger patient cohort are needed to evaluate the rate of recurrence of GP after the above procedure, along with the frequency of VKC exacerbations. Being a chronic disease, there is a likelihood of exacerbations in the long-term, and a longer follow-up will help to achieve a true idea of the efficiency of the above treatment.

## Figures and Tables

**Table 1:** Shows patient demographics, pre- and postoperative visual acuity, corneal complications, and follow-up duration.

Case	Age	Gender	VKC Duration (Years)	Initial BCVA		Corneal Complications	Final BCVA		Follow-Up Duration Post-Op (Months)
				RE	LE		RE	LE	
1	7	M	4	6/15 #	6/12	Diffuse epitheliopathy	6/6 #	6/7.5	77
2	11	M	4	6/24 *	6/9	Shield ulcer	6/15 *	6/15	70
3	7	M	8	6/9 *	6/9 *	Shield ulcer	6/7.5 *	6/7.5 *	RE-66 LE-63
4	14	M	5	6/9 *	6/12	Shield ulcer	6/9.5 *	6/12	59

(BCVA: Best Corrected Visual Acuity); # eye that underwent surgical excision of giant papillae with MMC; * eyes that underwent surgical excision of giant papillae with MMC with AMT.

**Table 2:** Shows type of surgeries performed in all patients.

Case	Eye	No. of Surgeries	Type of Surgery
1	RE	3	(1) Right papillectomy 4/3/2011 (2) Right papillectomy with MMC 6/1/2012 (3) Right papillectomy with MMC 18/10/2013
2	RE	4	(1) Right papillectomy with MMC 22/2/2013 (2) Right papillectomy with MMC 29/11/2013 (3) Right papillectomy with MMC and supratarsal triamcinolone 23/5/2014 (4) Right papillectomy with MMC and AMT 19/6/2015
3	RE	3	(1) Right papillectomy with MMC 9/1/2015 (2) Right papillectomy with MMC 13/3/2015 (3) Right papillectomy with MMC and AMT 16/9/2015
	LE	2	(1) Left papillectomy with MMC and AMT 21/8/2015 (2) Left papillectomy with MMC and AMT 14/12/2015
4	RE	1	(1) Right papillectomy with MMC and AMT 22/7/2016

MMC—Injection Mitomycin C 0.02%; AMT—Amniotic Membrane Transplantation.

**Table 3:** Shows various surgical procedures with their outcomes in refractory VKC patients.

Study	Type of Study	Surgery Performed	No of Eyes	Age Group	Follow Up	Outcome
Cameron, J.A., 1995 [22]	Retrospective case series	Scrapping of base and margin of ulcer	23	5 to 19 years	0.5 week to 15.7 months	Rapid re-epithelization was seen in 20 of 23 ulcers and plaques
Nishiwaki -Dantas, M.C. et al., 2000 [23]	Retrospective case series	Giant papillary resection with free autologous inferior conjunctival graft	6	7 to 14 years	9 to 27 months	Recurrence of papillae was observed in only one patient (16.6%)
Tanaka, M. et al., 2004 [24]	Retrospective case series	Simple papillary resection	17	6 to 28 years	12–18 months	Papillary regrowth—84.2% of cases, Recurrence of corneal leisons—47.1%
		Giant papillary resection with intraoperative MMC 0.02%	7	7 to 16 years	12–18 months	Papillary regrowth—57.1% of cases, Recurrence of corneal leisons—14.3%
Jiang, D. et al., 2006 [25].	Retrospective case series	Giant papillary resection with cryotherapy with AMT	16	8 to 16 years	3–22 months	Incidence of GP recurrence was 12.5%
Guo, P. et al., 2013 [26]	Retrospective case series	Giant papillary resection with AMT	13	7 to 37 years	9 to 22 months	No recurrence of GP.

## Data Availability

All data generated or analyzed during this study are included in this published article.
